# Investigation on Gold Dissolution Performance and Mechanism in Imidazolium Cyanate Ionic Liquids

**DOI:** 10.3390/molecules29040897

**Published:** 2024-02-18

**Authors:** Na Zhang, Yuxin Zhang, Zhengyu Liu, Ziyuan Liu, Chunbao Sun, N. Emre Altun, Jue Kou

**Affiliations:** 1 School of Civil and Resource Engineering, University of Science and Technology Beijing, Beijing 100083, China; 2 Department of Mining Engineering, Middle East Technical University, Ankara 06800, Türkiye

**Keywords:** imidazolium cyanate, ionic liquid, gold dissolution, *N*-heterocyclic carbene

## Abstract

To explore green gold leaching reagents, a series of imidazolium cyanate ionic liquids (ILs), 1-ethyl-3-methyl-imidazolium cyanate ([C_2_MIM][OCN]), 1-propyl-3-methyl-imidazolium cyanate ([C_3_MIM][OCN]) and 1-butyl-3-methyl-imidazolcyanate ([C_4_MIM][OCN]) were synthesized and characterized by Nuclear Magnetic Resonance (NMR), Fourier Transform Infrared Spectroscopy (FTIR) and thermogravimetric (TG) analysis. In this research, the imidazolium cyanates were utilized as a solute, which not only decreased the usage of ILs but also increased their gold dissolution capability. The gold dissolution performances of three imidazolium cyanates were characterized by dynamic leaching test and Scanning Electron Microscopy (SEM). The results show that the all three imidazolium cyanates had a gold dissolution ability, and the shorter the carbon chain on the imidazole ring in imidazolium cyanate, the faster the gold dissolution rate. The gold dissolution performance of [C_2_MIM][OCN] was the best, and the weight loss of gold leaf was 2.9 mg/cm^2^ at 40 °C after 120 h dissolution in [C_2_MIM][OCN] mixed with 10 wt. % water. Besides this, the gold dissolution rate increased with the increase in the concentration of imidazolium cyanates as well as the reaction temperature. The gold dissolution performances of imidazolium cyanates in different solvents including water, acetonitrile, dimethyl sulfoxide (DMSO) and dimethylformamide (DMF) were also investigated, and the weaker the polarity of the solvent, the more conducive it was to the gold dissolution reaction. The mechanism of gold dissolution by imidazolium cyanates was investigated through NMR spectroscopy and Electrospray Ionization Mass Spectrometry (ESI-MS). It was inferred that during the process of gold dissolution, Au was oxidized to Au^+^ and the imidazolium cations were deprotonated to form *N*-heterocyclic carbenes, which coordinated with gold to form gold complexes and achieve gold dissolution.

## 1. Introduction

Gold is mostly obtained through leaching operations from Au-bearing minerals using cyanide salts [1]. Lately, with the development of the electronic industry, the recovery of gold from electronic waste has also gained attention [2]. The traditional gold leaching method is cyanidation. However, cyanide is highly toxic, and it not only threatens the user’s safety but also entails a high transportation risk [3,4]. The other leaching reagents such as thiourea, thiosulfate and chlorine all have their own advantages and disadvantages [5]. Up to now, there has been no reagent that can completely substitute cyanide to leach gold. To address environmental restrictions and for a non-toxic leaching and hydrometallurgical extraction of target values, efforts towards the development of and investigations into green, environmentally sound gold leaching reagents have increased, particularly over the last two decades.

Ionic liquids (ILs) are compounds completely composed of ions with melting points below 100 °C, which exhibit “green” and “designer” properties [6]. Ionic liquids are nonvolatile and nonflammable and can serve as effective substitutes for traditional organic solvents [7]. Ionic liquids have drawn attention in organic synthesis, metal electrodeposition, capacitors, catalysis, extraction and nanomaterial preparation [8,9]. Recently, research on the dissolution and extraction of metals in ionic liquids has drawn attention, and the dissolution of metals such as Cu, Ni, Fe and Al, and their oxides, in ionic liquids as the solvent has been reported [10,11,12]. In the gold extraction field, a 1-butyl-3-methyl-imidazolium hydrogen sulfate ionic liquid (bmimHSO_4_) water solution was mixed with thiourea and iron(III) sulphate to selectively extract Au from gold-bearing ore [13,14,15,16]. However, the existing reports have focused on the effects of ionic liquid on gold leaching by thiourea, and the reports on gold leaching using ionic liquid itself are quite limited.

Owing to the acidity of the C2–H group of imidazolium ionic liquids, imidazolium salts have the potential to be deprotonated and generate *N*-heterocyclic carbenes (NHCs), which are powerful ligands [17]. Carbenes possess a lone pair of electrons and a vacant orbital, and exhibit Lewis acidic and Lewis basic properties; therefore, they have a strong σ-donating ability [18]. An electrochemical methodology as well as suitable bases can both be utilized to deprotonate imidazolium salts and obtain NHCs [19,20]. The electrochemical properties of imidazolium-based ionic liquids have been studied, and the formation of stable NHCs in the imidazolium salt aqueous solution was detected with the assistance of water [21]. Since carbene can strongly bind metal centers of high and low oxidation states, it can combine with Au^+^ to form a carbene gold(I) complex [22,23,24,25]. Therefore, carbene has the potential to dissolve gold in suitable liquid conditions. Cyanate (NCO^−^ or OCN^−^) anions can also work as bridging ligands for transition metal complexes with Ag, Cu, Cr, Re, etc. [26,27]. Cyanate anion ionic liquid was first reported in 2011, and it turned out that [C_2_MIM][OCN] could facilitate gold dissolution [28]. Therefore, the combination of imidazolium cation and cyanate anion possesses the ability to dissolve gold. However, the gold dissolution mechanism of imidazolium cyanate ionic liquid is still unclear.

**Figure 1:**
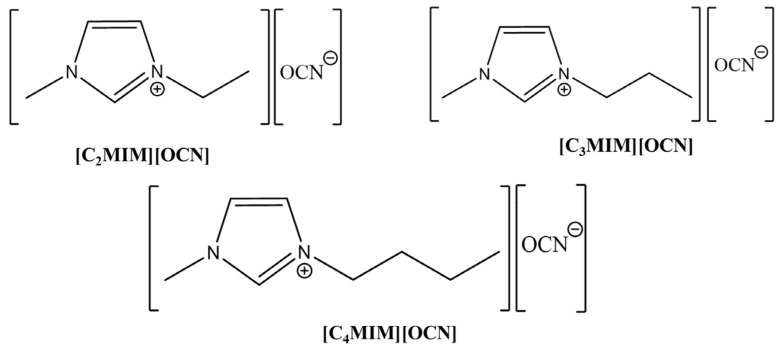
Chemical structures of the three imidazolium cyanate ionic liquids in this study.

In this study, three imidazolium cyanate ionic liquids, 1-ethyl-3-methylimidazolium cyanate ([C_2_MIM][OCN]), 1-propyl-3-methylimidazolium cyanate ([C_3_MIM][OCN]) and 1-butyl-3-methylimidazolium cyanate ([C_4_MIM][OCN]), were synthesized and characterized, and their chemical structures are shown in Figure 1. The dose of [C_4_mim]^+^ at which 50% of rats die (LD_50_) is reported to be approximately 550 mg kg^−1^, and the longer the alkyl chain length of the cation, the more toxic the ionic liquids are, so the toxicities of [C_2_mim]^+^ and [C_3_mim]^+^ were less than that of [C_4_mim]^+^ [29]. The LD_50_ of NaCN is reported as 3 mg kg^−1^ (Encyclopedia of Toxicology [30]). Therefore, the imidazolium cyanate ionic liquids synthesized could be called low-toxicity and green reagents. The imidazolium cyanates were mixed with different solvents to reduce their usage as well as to increase gold dissolution performance. The influences of temperature, concentration and solvent type on their gold dissolution rate were investigated. Besides this, the mechanism of gold dissolution by imidazolium cyanates was explored using NMR spectroscopy and ESI-MS.

## 2. Results and Discussion

### 2.1. Characterization of the Imidazolium Cyanate Ionic Liquid

**Figure 2:**
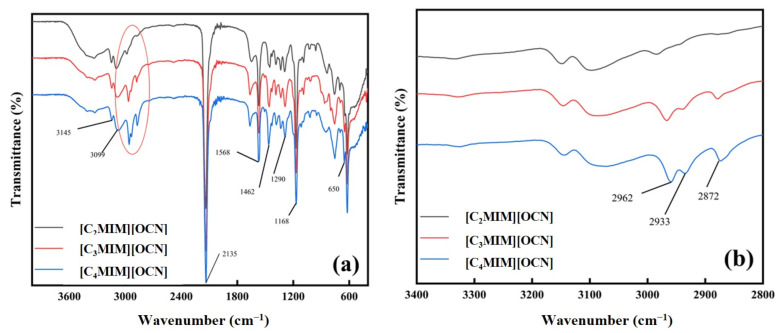
(**a**) FTIR spectra of imidazolium cyanate ionic liquids. (**b**) Magnification of the spectra highlighted in the oval in (**a**).

The synthesized imidazolium cyanate ionic liquids were characterized by NMR (Figure S1). FTIR spectroscopy supported the chemical structural information of the synthesized ionic liquids, especially the alkyl chain information. The FTIR spectra of the three imidazolium cyanate ionic liquids are shown in Figure 2. The absorption peaks near 3099 and 3145 cm^−1^ were C–H stretching vibrations of imidazolium cations, while the absorption peaks in the range of 2700~3000 cm^−1^ represent the C–H stretching vibrations of methyl and methylene groups. The absorption peak near 1568 cm^−1^ was attributed to the vibration of the imidazole ring, and the absorption peak near 1462 cm^−1^ was the deformation vibration of methyl C–H. The peak at 1168 cm^−1^ was due to the stretching vibration of the imidazole ring. The absorption peaks in the range of 500~1000 cm^−1^ were mainly generated by C–H in-plane bending vibrations. The characteristic peaks of the cyanate anion in the ionic liquid appeared near 2135, 1290 and 650 cm^−1^. These three absorption peaks represent the stretching vibration of C≡N, the bending vibration of C–O and the doubly degenerate bending vibration of OCN^−^ [31].

The FTIR spectra of the three ionic liquids were the same except the peaks near 2900 cm^−1^. This was due to the different lengths of alkyl chains in the three ionic liquid cations. Figure 2b shows a magnification of the FTIR peaks around 2900 cm^−1^, showing that the FTIR peaks of [C_4_MIM][OCN] at 2872 cm^−1^ and 2933 cm^−1^ were significantly more intense than those of the other two ionic liquids. There was no characteristic peak of carbonate at 2343 cm^−1^ in the spectrogram, indicating that cyanate did not undergo hydrolysis during the synthesis of the ionic liquid and was completely retained [28]. In addition, a small O–H absorption band appeared in the vicinity of 3400 cm^−1^, which was due to residual moisture in the ionic liquid.

**Figure 3:**
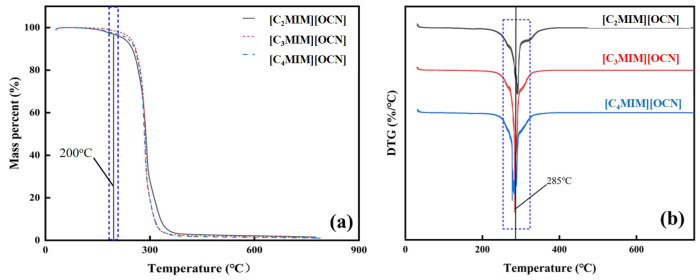
The TG (**a**) and DTG (**b**) curves for imidazolium cyanate ionic liquids. The temperature where the mass of IL started to change was labeled by the black line and emphasized in the blue dash rectangle.

The TG and DTG curves of the cyanate ionic liquids are shown in Figure 3. As seen from the result, the slope of the TG curve was small in the range of 30 to 200 °C, and the mass loss of the ionic liquids was less than 2 wt. %, which was caused by the evaporation of water in the ILs. This shows that the ionic liquid was not volatile and did not decompose. When the temperature exceeded 200 °C, the TG curve began to decrease and the slope increased, and the mass loss of the ionic liquid increased as well, indicating the initiation of a decomposition reaction. The DTG curves show that, in the whole temperature range, the three ionic liquids had a peak near 285 °C. The mass changes of the three ionic liquids were basically the same. The thermal decomposition of imidazolium cations was mainly due to the rupture of C–N bonds between the imidazole nitrogen and the adjacent alkyl chain carbon [32], so the type of anion played a decisive role in the thermal stability of ionic liquids [33].

### 2.2. The Gold Dissolution Performance of Imidazolium Cyanate Ionic Liquid

**Figure 4:**
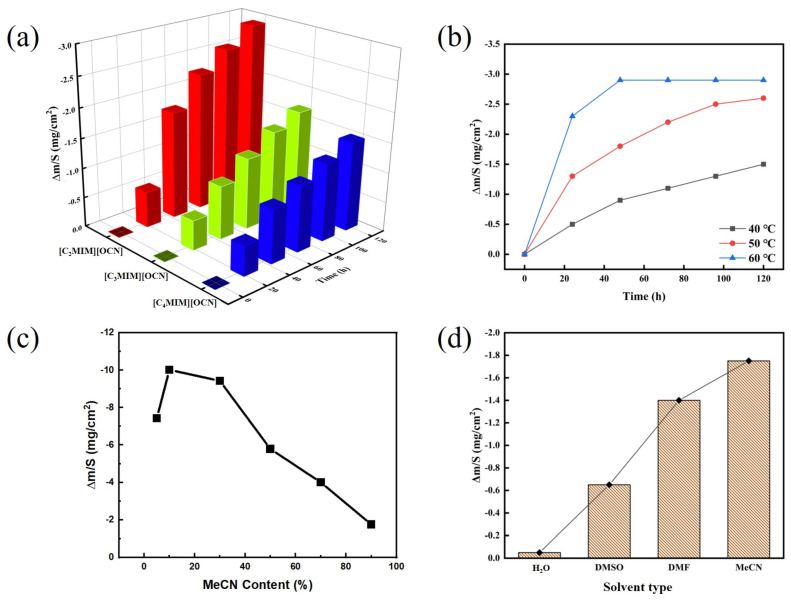
(**a**) Weight loss of gold leaf with time in different imidazolium cyanate ionic liquids with 10 wt. % water at 40 °C. (**b**) Weight loss of gold leaf with time in [C_4_MIM][OCN] with 10 wt. % water at different temperatures. (**c**) Weight loss of gold leaf in [C_4_MIM][OCN] mixed with different amounts of MeCN for 24 h at 60 °C. (**d**) Weight loss of gold leaf in [C_4_MIM][OCN] (10 wt. %) mixed with different solvents for 24 h at 60 °C.

To investigate the gold dissolution performance of the imidazolium cyanate ionic liquid, the gold dissolution abilities of three imidazolium cyanates were compared, and the effect of dissolution temperature was studied. Besides this, the gold dissolution capability of imidazolium cyanate mixed with different amounts of water was explored. The influences of different solvents on their gold dissolution capability were investigated as well. The results are shown in Figure 4.

The gold dissolution results of three imidazolium cyanates are shown in Figure 4a. For all three imidazolium cyanates, the weight losses of gold leaf all increased with time from 0 to 120 h. However, the weight loss of gold leaf was the largest in [C_2_MIM][OCN], at 2.9 mg/cm^2^ after 120 h dissolution. The dissolution rate of gold decreased with increasing imidazolium cationic alkyl substituents. The measured viscosities were 2.3 cP, 4.7 cP and 7.8 cP for [C_2_MIM][OCN], [C_3_MIM][OCN] and [C_4_MIM][OCN] mixed with 10% water, respectively. With the increase in alkyl chain length, the viscosity of the ionic liquid increased, which reduced the mass transfer rate of the reaction [32]. The gold dissolution rate was retarded by its slow diffusion from the surface into the solution. In addition, with the increase in alkyl chain length, the steric hindrance increased, which also hindered the reaction rate.

**Figure 5:**
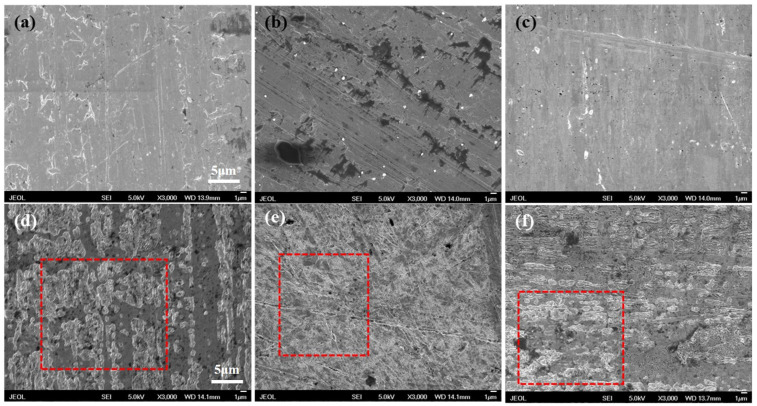
SEM images of gold leaf before (**a**–**c**) and after (**d**–**f**) dissolution in [C_2_MIM][OCN] (**d**), [C_3_MIM][OCN] (**e**) and [C_4_MIM][OCN] (**f**) mixed with water (10 wt. %) for 24 h at 40 °C. The scale bar for all micrographs is 5 μm. The typical dissolved areas were labeled by red dash rectangles.

The morphology of gold leaf before and after dissolution in imidazolium cyanate ionic liquids is shown in Figure 5. The surfaces of gold leaves before dissolution (Figure 5a–c) were relatively smooth except for some scratches and polishing marks. After dissolution in [C_2_MIM][OCN], [C_3_MIM][OCN] and [C_4_MIM][OCN], the surfaces of gold leaves (Figure 5d–f) were corroded and became rough. As seen in the area labeled in the red square (Figure 5d–f), the corrosion started from the defects on the gold leaves. It can be ascertained from the degree of corrosion that the gold dissolution ability of [C_2_MIM][OCN] ranked the highest among the three imidazolium cyanate ionic liquids. The effect of temperature on the gold dissolution rate of [C_4_MIM][OCN] is shown in Figure 4b. The weight loss of gold leaf with time increased at 40 °C, 50 °C and 60 °C. Obviously, the dissolution rate increased with the increase in temperature. The weight loss of gold leaf increased from 0.5 mg/cm^2^ to 2.3 mg/cm^2^ after 24 h, when the temperature increased from 40 °C to 60 °C. The dissolution reached a balance after 48 h at 60 °C, and then the weight loss of gold leaf barely changed. The increase in temperature greatly increased the average kinetic energy of molecules and promoted the mass transfer rate. Moreover, the self-diffusion coefficient of the anions of the ionic liquid in aqueous solution systems was significantly improved at 60 °C, which increased the probability of molecular collision and bonding, so the gold dissolution rate was accelerated.

The concentration of imidazolium cyanates affected the dissolution rate of gold leaf, and the result for [C_4_MIM][OCN] mixed with different amount of MeCN at 60 °C is illustrated in Figure 4c. After 24 h of dissolution, the weight loss of gold leaf was 7.4 mg/cm^2^ in pure [C_4_MIM][OCN] ionic liquid. With the addition of 10% MeCN, the gold dissolution rate increased to 10.0 mg/cm^2^. When the MeCN content increased, the change in gold leaf quality decreased. The addition of 10% MeCN decreased the viscosity of IL, which increased the mass transfer rate in the solution and improved the reaction rate. However, the overdose of MeCN diluted the imidazolium cyanates, which decreased the dissolution rate.

The weight loss of gold leaf was ~1.7 mg/cm^2^ when 90% MeCN was added in [C_4_MIM][OCN], which is comparable to that when 10% water was added in [C_4_MIM][OCN]. Since MeCN affected the gold dissolution rate of imidazolium cyanates, solvent type might influence it as well. Therefore, the gold dissolution rates of [C_4_MIM][OCN] (10 wt. %) in water, dimethyl sulfoxide (DMSO), *N*,*N*-dimethyl formamide (DMF) and acetonitrile (MeCN) for 24 h at 60 °C were compared. The results are shown in Figure 4d. The results in [C_4_MIM][OCN] with 10% solvent are shown in Figure S3, which had the same tendency as those shown in Figure 4d. The weight loss of gold leaf in [C_4_MIM][OCN] mixed with H_2_O hardly changed, while in the organic solvents, the gold dissolution rate increased in the order of DMSO < DMF < MeCN. Namely, the dissolution rate increased with the decrease in solvent polarity (acetonitrile was the least polar). Water was a strongly polar protic solvent that can provide protons by itself [34]. Acetonitrile and the other three organic solvents were less polar than aqueous solutions and had weaker proton supply abilities, which was conducive to the self-deprotonation of imidazolium cations [35,36]. Therefore, the weaker the polarity of the solvent, the more conducive to the gold dissolution reaction.

### 2.3. Investigation into the Gold Dissolution Mechanism of Imidazolium Cyanate Ionic Liquid

**Figure 6:**
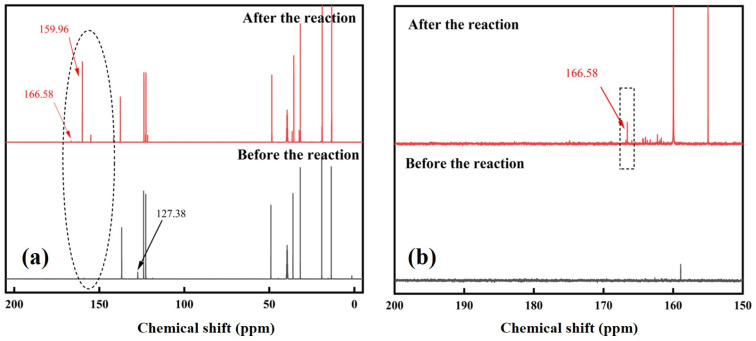
(**a**) ^13^C NMR spectra of [C_4_MIM][OCN] before and after gold dissolution in [C_4_MIM][OCN] mixed with H_2_O (10 wt. %) at 60 °C for 24 h. (**b**) Magnification of the spectra shown in the oval in (**a**). The NMR difference before and after the reaction was labeled in the black dash rectangle.

To investigate the gold dissolution mechanism related to imidazolium cyanates, the NMRs of dissolution solution before and after dissolution were compared. The pregnant solution was collected and detected by NMR after the Au reacted with [C_4_MIM][OCN] mixed with H_2_O (10 wt. %) at 60 °C for 24 h. Compared with the ^13^CNMR spectra of the ionic liquid before reaction, after dissolution, the chemical shift of C in each structure of imidazolium cyanate did not change significantly. However, the C peak of cyanate at 127.38 ppm disappeared (Figure 6). At the same time, several new C peaks appeared between 150 and 170 ppm. The C peak at 159.96 ppm was carbonate generated by the hydrolysis of cyanate in the aqueous system. Cyanate groups in the aqueous system would be hydrolyzed, leading to the disappearance of the cyanate peak after dissolution [37,38], which supports the use of an alkaline environment for the generation of *N*-heterocyclic carbene. There was a weak C signal peak at 166.58 ppm, which is related to the chemical shift of the C2 peak on the imidazole ring in *N*-heterocyclic carbene gold complexes [39].

**Figure 7:**
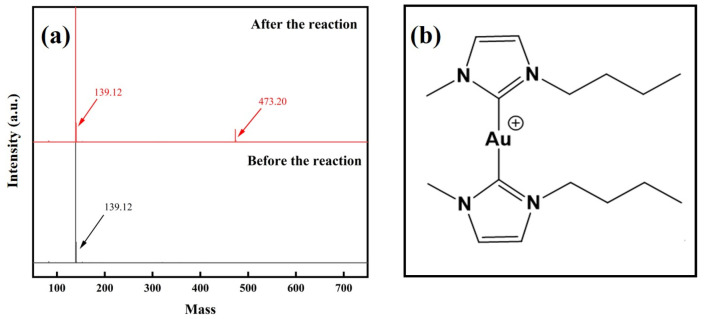
(**a**) ESI-MS spectra of [C_4_MIM][OCN] before and after gold dissolution in [C_4_MIM][OCN] mixed with H_2_O (10 wt. %) at 60 °C for 24 h. (**b**) The structure of the gold complex (*m*/*z* 473.20) generated via gold dissolution by [C_4_MIM][OCN].

To further investigate gold dissolution using an imidazolium cyanates ionic liquid, The ESI-MS values of the pregnant solution before and after gold dissolution in [C_4_MIM][OCN] mixed with H_2_O (10 wt. %) at 60 °C for 24 h were compared. As seen in Figure 7a, a strong signal peak appeared at *m*/*z* 139.12 before and after the reaction in ionic liquid, which was the signal peak of [C_4_MIM]^+^ cations. After gold dissolution, a new signal peak appeared in the positive spectrum, indicating that a new substance with a mass to charge ratio of 473.20 was generated. Combined with the NMR results, we can infer that the new substance produced was a cationic complex formed by the combination of two carbene ligands and a gold ion (Figure 7b). The ESI-MS values of the pregnant solution before and after gold dissolution in [C_2_MIM][OCN] mixed with H_2_O (10 wt. %) at 60 °C for 24 h are shown in Figure S2, which also supports the generation of a gold complex made of two NHC and one Au^+^.

Based on the results in Figure 6 and Figure 7, the reaction of gold in the imidazolium cyanate solution could be inferred. The Au could be oxidized by O_2_ when the Au^+^ forms a complex with ligands, such as CN^−^, S_2_O_3_^2−^ and Cl^−^ [40]. Since a gold complex was detected in the product (Figure 7b), Au was theorized to have been oxidized to Au^+^ by O_2_, as in Equation (1):

(1) $$4Au+O_{2}+{2H}_{2}O\to 4{Au}^{+}+4{OH}^{-}$$

The cyanate anions provided an alkaline environment for the imidazolium cations. The H at C2 on imidazole was highly acidic, and could be deprotonated under alkaline conditions. After deprotonation, *N*-heterocyclic carbenes (NHCs) were formed with a lone electron pair at the C2 position, as shown in Equation (2):

(2) $$[C_{4}MIM{]}^{+}+{OH}^{-}\overset{[OCN{]}^{-}}{\to }\left[C_{4}MIM\right](NHC)+H_{2}O$$

NHCs had a strong σ donor and a weak π receptor, and served as an excellent ligand for Au^+^. When the lone electron pair in the atom was hybridized with the vacant orbital of the Au^+^, the original electron cloud density distribution around the atom changed and a gold complex was formed, as shown in Equation (3):

(3) $${Au}^{+}+2NHC\to Au(NHC{)}_{2}^{+}$$

**Figure 8:**
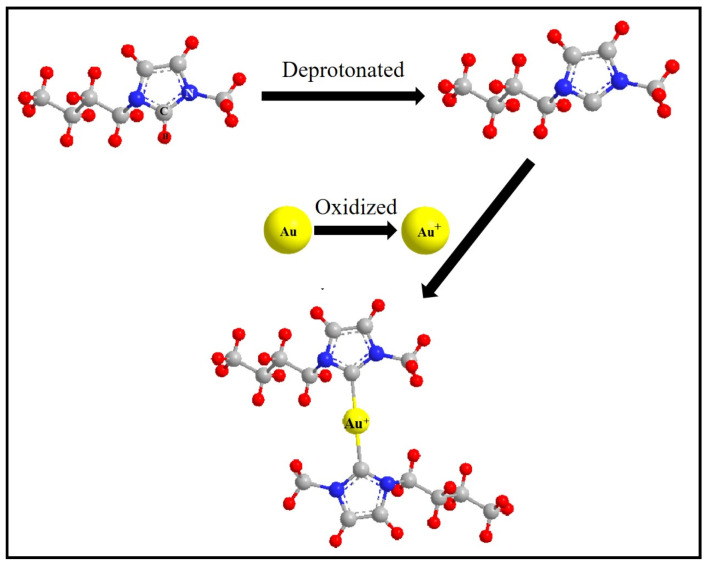
Schematic of the reaction between Au and imidazolium cations.

Combining the NMR and ESI-MS results, the process of gold dissolution in imidazolium cyanate ionic liquid was inferred, as shown in Figure 8. The C2 position H of the imidazole ring was deprotonated, and a *N*-heterocyclic carbene was formed. Au was oxidized into Au^+^, which coordinated with two NHC ligands to form gold carbene complexes. The gold was dissolved in this process.

## 3. Materials and Methods

### 3.1. Materials and Reagents

The information regarding the chemicals and materials used is shown in Table 1. The water used in this study was deionized with a conductivity of 0.054 µS/cm.

### 3.2. The Synthesis of Imidazolium Cyanate Ionic Liquids

**Figure 9:**
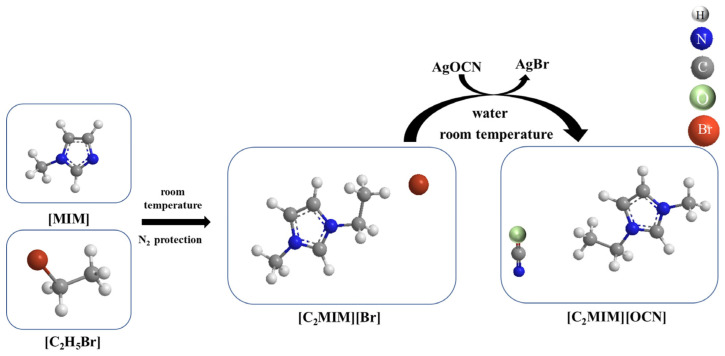
Schematic of the synthesis of 1-ethyl-3-methylimidazolium cyanate.

The procedure followed to synthesize the imidazolium cyanate ionic liquids is shown in Figure 9 [28]. Here, [C_2_MIM][OCN] was taken as the example to describe the synthesis process, and the synthesis parameters for [C_3_MIM][OCN] and [C_4_MIM][OCN] were the same as those for [C_2_MIM][OCN], except that bromoethane was replaced with bromopropane and with bromobutane as the reactant, respectively. The first step was to synthesize imidazolium bromide ([C_2_MIM][Br]) using bromoethane and *N*-ethylimidazole. Then, silver cyanate (AgOCN) was synthesized. Lastly, [C_2_MIM][OCN] was obtained from the reaction between [C_2_MIM][Br] and AgOCN.

#### 3.2.1. Synthesis of 1-Ethyl-3-methylimidazolium Bromide

Bromoethane and *N*-methylimidazole with a molar ratio of 1.2:1 were put in a round-bottom flask and protected with nitrogen to initiate a cooling reflux reaction. When white crystals were no longer produced, 100 mL of acetonitrile and ethyl acetate mixture (1:1 in volume) were added. The solution was heated to 70 °C, and the crystals were melted in the solution. After 20 min of the cooling reflux reaction, the product was cooled to room temperature. Following the complete precipitation of white crystalline 1-ethyl-3-methylimidazolium bromide ([C_2_MIM][Br]), vacuum filtration was performed. After being washed and filtered, the [C_2_MIM][Br] crystals were transferred to a vacuum dryer to remove residual solvents. The reaction equation is shown in Equation (4):
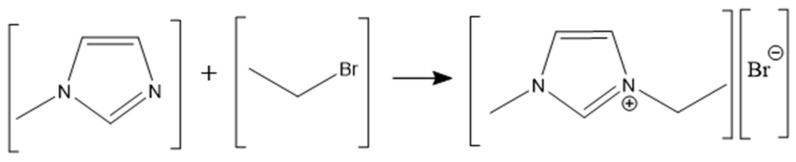
(4)

#### 3.2.2. Synthesis of Silver Cyanate

Here, 14.30 g sodium cyanate was dissolved in 200 mL water and 33.97 g silver nitrate was dissolved in 200 mL water. Then, the two solutions were mixed and stirred for 12 h at room temperature. White precipitates were generated and filtered, and then were washed with 100 mL water. Finally, the product was dried under high vacuum for 24 h, and silver cyanate was prepared. The reaction equation is shown in Equation (5):

AgNO_3_ + NaOCN → AgOCN + NaNO_3_ (5)

#### 3.2.3. Synthesis of 1-Ethyl-3-methylimidazole Cyanate

[C_2_MIM][Br] (11.464 g, 0.06 mol) and AgOCN (8.99 g, 0.06 mol) were mixed with 300 mL water and the reaction was carried out at room temperature under dark conditions for 12 h. Yellow precipitates were generated and filtered. The filtrate was placed in a refrigerator to rest (0 °C) for 12 h before being filtered again. The solvent was removed by vacuum distillation and the process was repeated twice with acetonitrile. Finally, the product was freeze-dried in vacuum to remove excess solvent, and 1-ethyl-3-methylimidazolium cyanate ([C_2_MIM][OCN]) was obtained with a yield of 95% [28]. The reaction equation was shown in Equation (6):
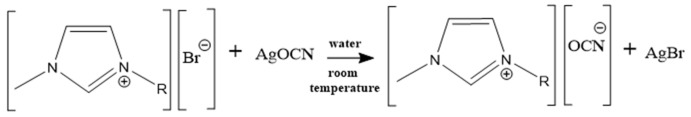
(6)

### 3.3. FTIR Spectroscopy

The FTIR spectrum was obtained by a Nicolet iS50 Fourier transform infrared spectrometer (Thermo Fisher Scientific, Waltham, MA, USA). The sample was scanned multiple times within the range of 400–4000 cm^−1^. The infrared spectrum of the target product was measured, and the chemical bonds and functional groups between different compounds were analyzed to judge the structure of the synthesized product and whether there were byproducts or impurities in the synthesis process.

### 3.4. TG-DTG Analysis

The thermal stability of the imidazolium cyanate ionic liquids was characterized by a STA8000 synchronous calorimeter (PerkinElmer, Waltham, MA, USA). Analysis was carried out in a 50 µL sealed alumina crucible with pinholes, and the flow rate of the protective gas argon was 20 mL/min. The sample was heated from 30 °C to 800 °C at a heating rate of 20 °C/min under the protection of argon, and the relationship curve between the weight loss of the ionic liquid and the temperature was obtained. The derivative thermogravimetric analysis (DTG) curve was the derivative of the TG curve.

### 3.5. Dissolution Test

**Figure 10:**
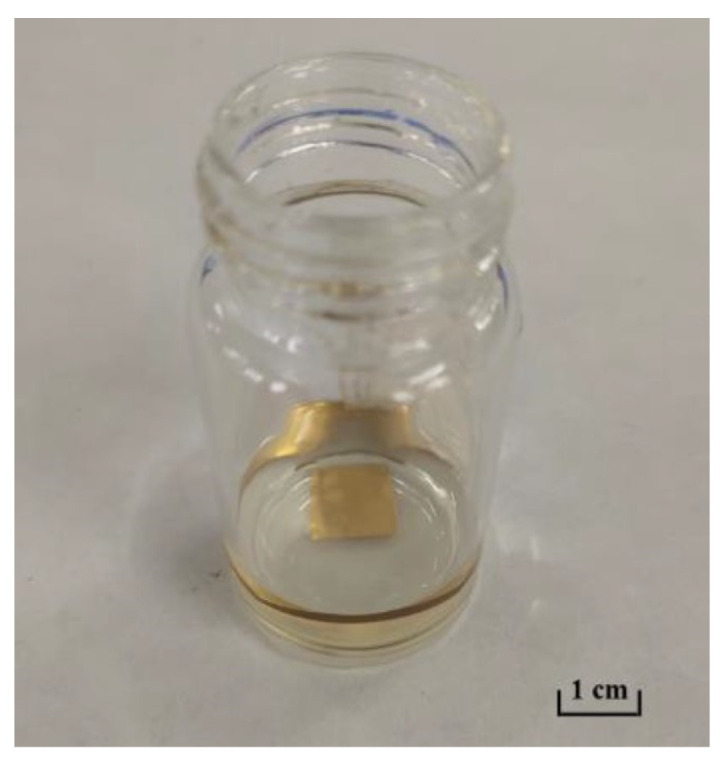
The dynamic gold dissolution test in a container.

The dynamic leaching tests were carried out in a container of 3 cm diameter and 5 cm height (Figure 10). The gold leaf (10 × 10 × 0.1 mm) used in the reaction was cleaned in diluted hydrochloric acid solution, acetone and deionized water in sequence via ultrasonication. The gold leaf was then weighed (to an accuracy of 0.0001) to record its initial mass. The imidazolium cyanate ionic liquid was mixed with solvent (water, acetonitrile, DMSO, DMF) with a mass ratio of 9:1, the pH of which was 12~13, measured using pH test paper. The viscosities of three imidazolium cyanates mixed with 10% water at 40 °C were detected using a Brookfield DV2EXTRA-Pro rotational viscometer (AMETEK Brookfield, Middleboro, MA, USA). The gold leaf was then placed in a glass bottle with 4 g imidazolium cyanate solution. The agitated dissolution tests were carried out in a thermostatic oscillator with a frequency of 100 rpm at different temperatures (40 °C, 50 °C, 60 °C). Different amounts of solvent were added to the [C_4_MIM][OCN] to investigate the effect of solvent content on its gold dissolution ability at 40 °C. The gold leaf was dried and weighed every 24 h for 120 h. The container was opened to make sure the O_2_ was dissolved in the solution.

### 3.6. Scanning Electron Microscopy Investigation

The surface morphology of gold leaf before and after IL dissolution was observed using a JSM-6701 cold field emission Scanning Electron Microscope (JEOL, Tokyo, Japan). The gold leaf was polished before dissolution, and then its surface morphology was identified; after the reaction with different imidazolium cyanate ionic liquids mixed with water (10 wt. %) for 24 h at 40 °C, the surface of the gold leaf was washed with deionized water, and then dried in a vacuum drying box for re-identification using SEM. The SEM images were taken at 5 kV accelerating voltage, 20 spotsize, and 14 mm working distance.

### 3.7. Nuclear Magnetic Resonance Spectroscopy

A nuclear magnetic resonance (NMR) spectrometer (Bruker, AVANCE III HD 600, Bremen, Germany) was employed to characterize the structure of the synthesized product. The magnetic field parameters were optimized and the sampling parameters were set to obtain the ^1^H NMR and ^13^C NMR spectra of the target product. MestReNOVA software 12.0.4 was used for spectral processing. Different peaks in the ^1^H NMR spectra were integrated, and chemical shifts of different peaks in the ^13^C NMR spectra were classified.

### 3.8. Electrospray Mass Spectrometry

The mass spectrometry analysis was performed on a microTOF-Q II mass spectrometer (Bruker, Waltham, MA, USA) using an electrospray ionization (ESI) source and full scan detection mode. The ion source temperature was 180 °C, and the nebulizer gas and desolvating gas were both N_2_. The air pressure and gas flow velocity were 0.8 bar and 4.0 L/min, respectively. The capillary voltage was 3.5 kV, and the scanning range was 50~750 *m*/*z*. Data analysis was performed using the Bruker ESI mass spectrometer data processing software DataAnalysis 4.3. Additionally, 0.2 g of ionic liquid was dissolved in acetonitrile, and a gold dissolution test was conducted at 60 °C. The reaction solution was collected and dried for ESI-MS mass spectrometry analysis.

## 4. Conclusions

In this study, three kinds of imidazolium cyanate ionic liquids, [C_2_MIM][OCN], [C_3_MIM][OCN] and [C_4_MIM][OCN], were synthesized and characterized with the goal of investigating and comparing their abilities to dissolve gold. The imidazolium cyanates were utilized as solute, which not only decreased the usage of ILs, but also increased the gold dissolution performance. The mechanism of gold dissolution by imidazolium cyanate was also explored. The main conclusions derived from this work are as follows:

(1) The synthesized imidazolyl cyanate ionic liquids ([C_2_MIM][OCN], [C_3_MIM][OCN] and [C_4_MIM][OCN]) were characterized by NMR, FTIR and TG-DTG analysis. The three ILs were stable when under 200 °C, and the water contents in the synthesized ILs were less than 2 wt. %;
(2) All three imidazolyl cyanate ionic liquids had a gold dissolution ability, and [C_2_MIM][OCN] showed the best performance because of its low viscosity and small steric hindrance. The increase in temperature promoted the gold dissolution rate by promoting the mass transfer rate. The increase in solvent content in the imidazolyl cyanate solution decreased its gold dissolution ability, for the ions in the ionic liquid was surrounded by H_2_O molecules, and this prohibited the gold-solubilizing process. The polarity of the solvent also affected the gold dissolution capability of imidazolyl cyanate, and the weaker the polarity of the solvent, the more conducive it was to the gold dissolution reaction;
(3) The mechanism of gold dissolution by imidazolium cyanate was investigated by NMR and ESI-MS. The mechanism of the gold dissolution reaction in imidazolyl cyanate solution was presented as a hypothesis. The gold dissolution in imidazolium cyanate [C_4_MIM][OCN] involved deprotonation in the C2 position of the imidazole ring and the forming of NHCs. Au was oxidized by oxygen to Au^+^ and combined with NHC ligands to form carbene gold complexes, which enabled the gold dissolution. However, the mechanism needs to be further explored.

The imidazolium cyanate solution showed the potential for use in recycling gold from electronic waste, wherein the form of Au is not as complicated as that in gold-bearing minerals. Besides this, the modified graphene showed the ability to recover gold from the IL solution. The application of imidazolium cyanate ILs in recycling gold from electronic waste will be further developed in future research.

## Figures and Tables

**Table 1:** Information of chemicals and materials used in this study.

Name	Chemical Formula	Specification	Application	Suppliers
Sodium cyanate	NaOCN	>97%	IL synthesis	Yuanye Biological Technology Co., Ltd. (Shanghai, China)
*N*-ethylimidazole	C_4_H_6_N_2_	Analytical grade	IL synthesis	Dibo Chemical Technology Co., Ltd. (Shanghai, China)
Silver nitrate	AgNO_3_	Analytical grade	IL synthesis	Tongguang Fine Chemical Company (Beijing, China)
Bromoethane	C_2_H_5_Br	Analytical grade	IL synthesis	Yinuokai Technology Co., Ltd. (Beijing, China)
Bromopropane	C_3_H_7_Br	Analytical grade	IL synthesis	Yinuokai Technology Co., Ltd.
Bromobutane	C_4_H_9_Br	Analytical grade	IL synthesis	Yinuokai Technology Co., Ltd.
Acetonitrile	CH_3_CN	Analytical grade	IL synthesis/Solvent	Yinuokai Technology Co., Ltd.
Ethyl acetate	CH_3_COOC_2_H_5_	Analytical grade	IL synthesis	Yinuokai Technology Co., Ltd.
Dimethyl sulfoxide (DMSO)	(CH_3_)_2_SO	Analytical grade	Solvent	Yinuokai Technology Co., Ltd.
Dimethylformamide (DMF)	HCON(CH_3_)_2_	Analytical grade	Solvent	Yinuokai Technology Co., Ltd.
Gold leaf (1 cm × 1 cm)	Au	>99.99%	Dissolution test	Yinuokai Technology Co., Ltd.

## Data Availability

Data are contained within the article and Supplementary materials.

## Supplementary Materials

The following supporting information can be downloaded at: https://www.mdpi.com/article/10.3390/molecules29040897/s1, Figure S1: (a) ^1^H NMR spectra of three imidazolium cyanate ionic liquids [C_2_MIM][OCN], [C_3_MIM][OCN] and [C_4_MIM][OCN]. (b) ^13^C NMR spectra of three imidazolium cyanate ionic liquids [C_2_MIM][OCN], [C_3_MIM][OCN] and [C_4_MIM][OCN]; Figure S2: (a) ESI-MS spectra of [C_2_MIM][OCN] before and after gold dissolution in [C_2_MIM][OCN] mixed with H_2_O (10 wt. %) at 60 °C for 24 h. (b) The structure of the gold complex (*m*/*z* 417.13) generated in gold dissolution by [C_2_MIM][OCN]; Figure S3: Weight loss of gold leaf in [C_4_MIM][OCN] mixed with different solvents (10 wt. %) for 24 h at 60 °C.

## References

[1] Aylmore M.G., Muir D.M. (2001). Thiosulfate leaching of gold—A review. Miner. Eng..

[2] Yue C., Sun H., Liu W.J., Guan B., Deng X., Zhang X., Yang P. (2017). Environmentally benign, rapid, and selective extraction of gold from ores and waste electronic materials. Angew. Chem..

[3] Sun C.-b., Zhang X.-l., Kou J., Xing Y. (2020). A review of gold extraction using noncyanide lixiviants: Fundamentals, advancements, and challenges toward alkaline sulfur-containing leaching agents. Int. J. Miner. Metall. Mater..

[4] Zhang X., Sun C., Xing Y., Kou J., Su M. (2018). Thermal decomposition behavior of pyrite in a microwave field and feasibility of gold leaching with generated elemental sulfur from the decomposition of gold-bearing sulfides. Hydrometallurgy.

[5] Zhang N., Kou J., Sun C. (2023). Investigation on Gold-Ligand Interaction for Complexes from Gold Leaching: A DFT Study. Molecules.

[6] Lei Z., Chen B., Koo Y.-M., MacFarlane D.R. (2017). Introduction: Ionic Liquids. Chem. Rev..

[7] Freemantle M. (2010). An Introduction to Ionic Liquids.

[8] Forsyth S.A., Pringle J.M., MacFarlane D.R. (2004). Ionic liquids—An overview. Aust. J. Chem..

[9] Cao F., Wang W., Wei D.-z., Liu W.-g. (2021). Separation of tungsten and molybdenum with solvent extraction using functionalized ionic liquid tricaprylmethylammonium bis(2,4,4-trimethylpentyl)phosphinate. Int. J. Miner. Metall. Mater..

[10] Uerdingen M., Treber C., Balser M., Schmitt G., Werner C. (2005). Corrosion behaviour of ionic liquids. Green Chem..

[11] Bardi U., Chenakin S.P., Caporali S., Lavacchi A., Perissi I., Tolstogouzov A. (2006). Surface modification of industrial alloys induced by long-term interaction with an ionic liquid. Surf. Interface Anal..

[12] Kim B.-K., Lee E.J., Kang Y., Lee J.-J. (2018). Application of ionic liquids for metal dissolution and extraction. J. Ind. Eng. Chem..

[13] Teimouri S., Potgieter J.H., Simate G.S., Dyk L.V., Dworzanowski M. (2020). Oxidative leaching of refractory sulphidic gold tailings with an ionic liquid. Miner. Eng..

[14] Whitehead J.A., Lawrance G.A., McCluskey A. (2004). ‘Green’ leaching: Recyclable and selective leaching of gold-bearing ore in an ionic liquid. Green Chem..

[15] Whitehead J.A., Zhang J., McCluskey A., Lawrance G.A. (2009). Comparative leaching of a sulfidic gold ore in ionic liquid and aqueous acid with thiourea and halides using Fe(III) or HSO_5_^−^ oxidant. Hydrometallurgy.

[16] Whitehead J.A., Zhang J., Pereira N., McCluskey A., Lawrance G.A. (2007). Application of 1-alkyl-3-methyl-imidazolium ionic liquids in the oxidative leaching of sulphidic copper, gold and silver ores. Hydrometallurgy.

[17] Chiarotto I., Feroci M., Inesi A. (2017). First direct evidence of N-heterocyclic carbene in BMIm acetate ionic liquids. An electrochemical and chemical study on the role of temperature. New J. Chem..

[18] Frey G.D., Dewhurst R.D., Kousar S., Donnadieu B., Bertrand G. (2008). Cyclic (Alkyl)(amino)carbene Gold(I) complexes: A Synthetic and Structural Investigation. J. Organomet. Chem..

[19] Feroci M., Chiarotto I., Inesi A. (2013). Electrolysis of ionic liquids. A possible keystone for the achievement of green solvent-catalyst systems. Curr. Org. Chem..

[20] Stang P.J., Fox D.P. (1977). Alkylidene carbene generation from tosylazoalkenes and silylvinyl triflates. J. Org. Chem..

[21] Jain P., Chaudhari V.R., Kumar A. (2019). Water-assisted stability of carbene: Cyclic voltammetric investigation of 1-ethyl-3-methylimidazolium ethylsulfate ionic liquid. Phys. Chem. Chem. Phys..

[22] Frémont P.d., Scott N.M., Stevens E.D., Nolan S.P. (2005). Synthesis and Structural Characterization of N-Heterocyclic Carbene Gold(I) Complexes. Organometallics.

[23] Gaillard S., Cazin C.S.J., Nolan S.P. (2012). N-Heterocyclic Carbene Gold(I) and Copper(I) Complexes in C-H Bond Activation. Acc. Chem. Res..

[24] Gaillard S., Slawin A.M., Nolan S.P. (2010). A N-heterocyclic carbene gold hydroxide complex: A golden synthon. Chem. Commun..

[25] Nahra F., Tzouras N.V., Collado A., Nolan S.P. (2021). Synthesis of N-heterocyclic carbene gold(I) complexes. Nat. Protoc..

[26] Vicente R., Escuer A., El Fallah M.S., Solans X., Font-Bardia M. (1997). Three new mononuclear nickel (II) cyanate and isocyanate compounds derived from macrocyclic ligands: [Ni(TMCY)(NCO)](ClO_4_), [Ni(*m*-CTH)(OCN)_1.5_(ClO_4_)_0.5_]. Inorganica Chim. Acta.

[27] Escuer A., Vicente R., El Fallah M.S., Solans X., Font-Bardía M. (1996). Structure and magnetic behaviour of the first singly bridged nickel cyanate chain and a new dinuclear complex: An approximation to the superexchange mechanism for the nickel pseudohalide system. J. Chem. Soc. Dalton Trans..

[28] Janikowski J., Forsyth C., MacFarlane D.R., Pringle J.M. (2011). Novel ionic liquids and plastic crystals utilizing the cyanate anion. J. Mater. Chem..

[29] Kuroda K. (2022). A simple overview of toxicity of ionic liquids and designs of biocompatible ionic liquids. New J. Chem..

[30] Burr S.A., Wexler P. (2024). Cyanide. Encyclopedia of Toxicology.

[31] Schultz P.W., Leroi G.E., Popov A.I. (1995). The Structure and Thermodynamics of Hydrogen Bonding Interactions of OCN- with Methanol, Formamide, and N-methylformamide. J. Am. Chem. Soc..

[32] Handy S.T. (2005). Room temperature ionic liquids: Different classes and physical properties. Curr. Org. Chem..

[33] Kosmulski M., Gustafsson J., Rosenholm J.B. (2004). Thermal stability of low temperature ionic liquids revisited. Thermochim. Acta.

[34] Zhong X., Fan Z., Liu Z., Cao D. (2012). Local Structure Evolution and its Connection to Thermodynamic and Transport Properties of 1-Butyl-3-methylimidazolium Tetrafluoroborate and Water Mixtures by Molecular Dynamics Simulations. J. Phys. Chem. B.

[35] Marx D., Tuckerman M.E., Hutter J., Parrinello M. (1999). The nature of the hydrated excess proton in water. Nature.

[36] Wijaya Y.P., Smith K.J., Kim C.S., Gyenge E.L. (2022). Hydrodeoxygenation of lignin related phenolic monomers in polar organic electrolyte via electrocatalysis in a stirred slurry catalytic reactor. Green Chem..

[37] Xu L., Chen W., Xiao J. (2000). Heck Reaction in Ionic Liquids and the in Situ Identification of N-Heterocyclic Carbene Complexes of Palladium. Organometallics.

[38] Herrmann W.A., Böhm V.P.W., Reisinger C.-P. (1999). Application of palladacycles in Heck type reactions. J. Organomet. Chem..

[39] Zhou Y., Chen W. (2007). Synthesis and Characterization of Square-Planar Tetranuclear Silver and Gold Clusters Supported by a Pyrazole-Linked Bis(N-heterocyclic carbene) Ligand. Organometallics.

[40] Jeffrey M.I., Linda L., Breuer P.L., Chu C.K. (2002). A kinetic and electrochemical study of the ammonia cyanide process for leaching gold in solutions containing copper. Miner. Eng..

