# Supplementary material for: Investigation on Gold Dissolution Performance and Mechanism in Imidazolium Cyanate Ionic Liquids
Source: Molecules. 2024 Feb 18;29(4):897. doi: 10.3390/molecules29040897 (PMC10891921; doi:10.3390/molecules29040897)
Supplement: Supplementary file 1 [file molecules-29-00897-s001.zip › molecules-2838909-supplementary.pdf]

Investigation on Gold Dissolution Performance and Mechanism in Imidazolium Cyanate Ionic Liquids

Na Zhang [1], Yuxin Zhang [1], Zhengyu Liu [1], Ziyuan Liu [1], Chunbao Sun [1], N. Emre Altun [2,*] and Jue Kou [1,*]

[1] School of Civil and Resource Engineering, University of Science and Technology Beijing, Beijing 100083, China
[2] Department of Mining Engineering, Middle East Technical University, Ankara 06800, Türkiye
* Correspondence: ealtun@metu.edu.tr (N.E.A.); koujue@ustb.edu.cn (J.K.)

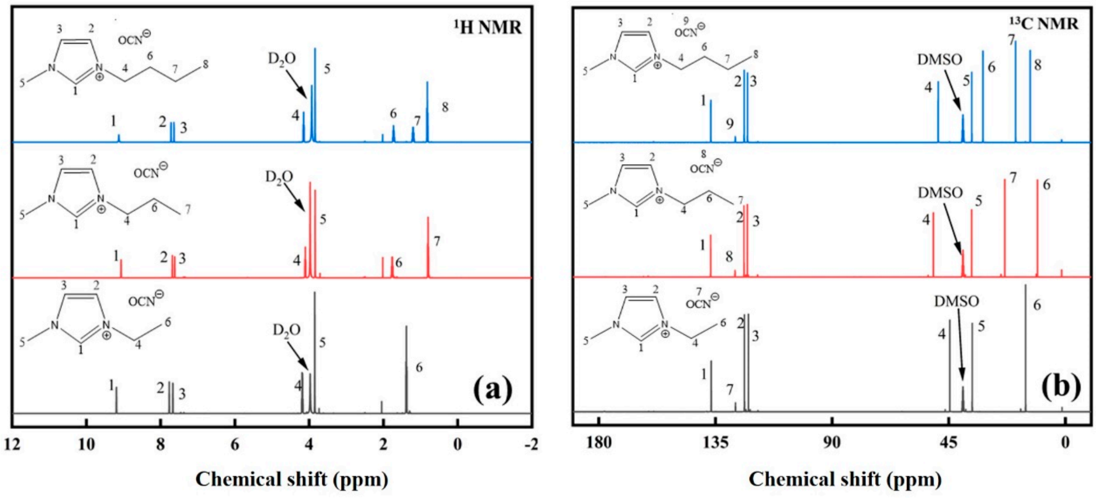

Figure S1 (a) ^{1}H NMR spectra of three imidazolium cyanate ionic liquids [C_2MIM][OCN], [C_3MIM][OCN] and [C_4MIM][OCN]. (b) ^{13}C NMR spectra of three imidazolium cyanate ionic liquids [C_2MIM][OCN], [C_3MIM][OCN] and [C_4MIM][OCN]

NMR investigation data.

[C_2MIM][OCN]: ^{1}H NMR (600 MHz, DMSO-d6): δ ppm = 9.19 (d, J = 1.7 Hz, 1H), 7.77 (t, J = 1.8 Hz, 1H), 7.67 (t, J = 1.8 Hz, 1H), 4.18 (q, J = 7.3 Hz, 2H), 3.85 (s, 3H), 1.38 (t, J = 7.3 Hz, 3H). ^{13}C NMR (151 MHz, DMSO-d6): δ ppm =136.64, 127.30, 123.83, 122.26, 44.59, 35.93, 15.35.

[C_3MIM][OCN]: ^{1}H NMR (600MHz，DMSO-d6): δ ppm = 9.07 (d, J = 1.7 Hz, 1H), 7.69 (t, J = 1.8 Hz, 1H), 7.62 (t, J = 1.8 Hz, 1H), 4.10 (t, J = 7.1 Hz, 2H), 3.84 (s, 3H), 1.76 (dt, J = 14.6, 7.3 Hz, 2H), 0.80 (t, J = 7.4 Hz, 3H). ^{13}C NMR (151 MHz, DMSO-d6): δ ppm = 136.86, 127.47, 124.01, 122.71, 50.91, 36.13, 23.37, 10.77.

[C_4MIM][OCN]: ^{1}H NMR (600MHz，DMSO-d6): δ ppm = 9.12 (s, 1H), 7.72 (d, J = 2.0 Hz, 1H), 7.64 (d, J = 2.0 Hz, 1H), 4.15 (t, J = 7.2 Hz, 2H), 3.84 (s, 3H), 1.77–1.69 (m, 2H), 1.25–1.15 (m, 2H), 0.82 (t, J = 7.4 Hz, 3H). ^{13}C NMR (151 MHz, DMSO-d6): δ ppm = 136.81, 127.38, 123.93, 122.66, 49.07, 36.10, 31.81, 19.18, 13.58.

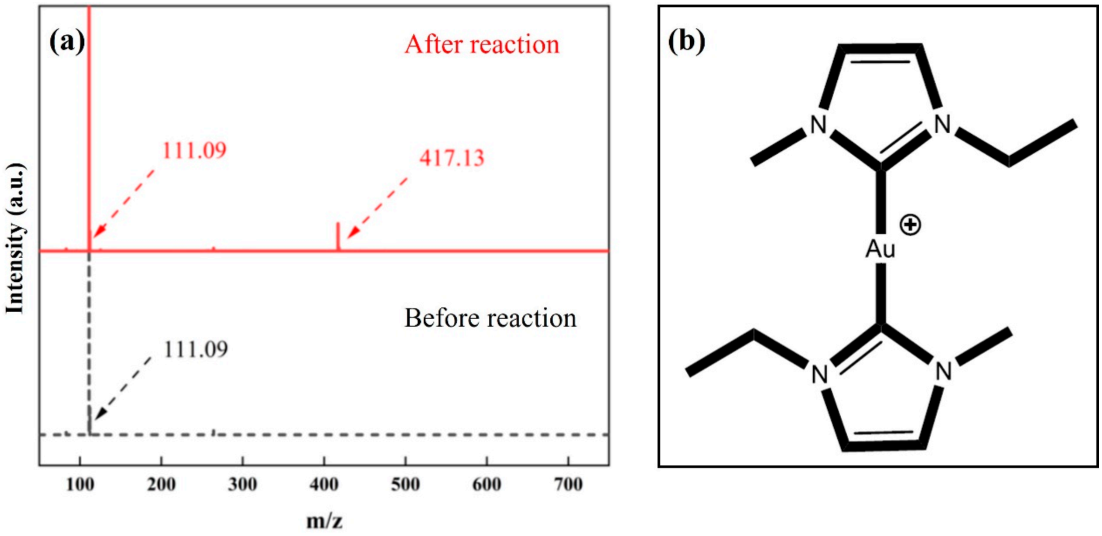

Figure S2. (a) ESI-MS spectra of [C_2MIM][OCN] before and after gold dissolution in [C_2MIM][OCN] mixed with H_2O (10 wt. %) at 60 °C for 24 h. (b) The structure of the gold complex (m/z 417.13) generated in gold dissolution by [C_2MIM][OCN].

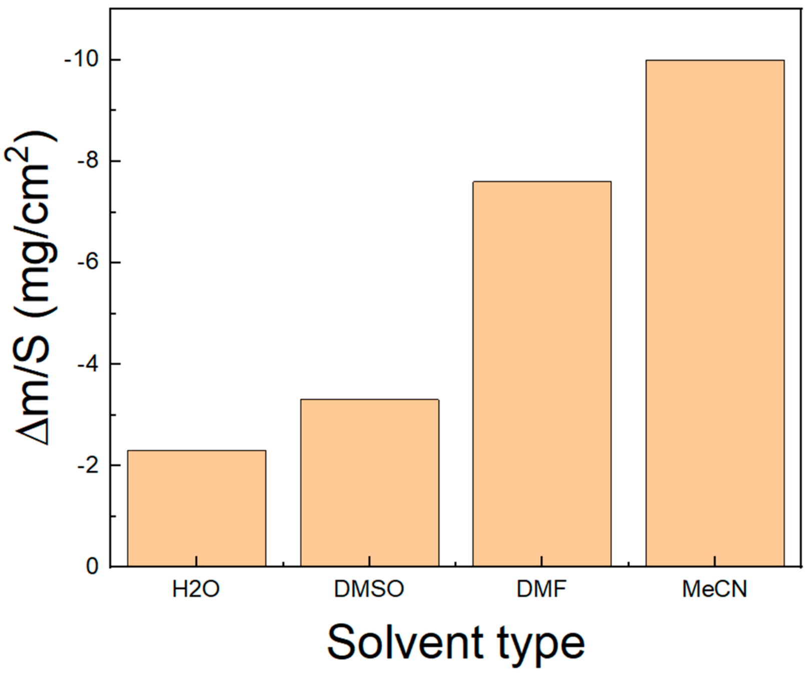

Figure S3. Weight loss of gold leaf in [C_4MIM][OCN] mixed with different solvents (10 wt. %) for 24h at 60 ^oC.
